# A broader look at Interceptive Orthodontics: What can we offer?

**DOI:** 10.1590/2177-6709.24.5.007-008.edt

**Published:** 2019

**Authors:** Flavia Artese

**Affiliations:** 1 Universidade do Estado do Rio de Janeiro, Departamento de Odontologia Preventiva e Comunitária (Rio de Janeiro/RJ, Brazil).

Preventive and interceptive orthodontics has the purpose of preventing or alleviating occlusal problems that might be happening in the transition period from deciduous to permanent dentition. In the spectrum of available procedures, we can include from the prevention of interproximal cavity, with the intention of maintaining arch length, all the way to two-phase orthodontic treatment, where the first phase is performed in the mixed dentition with the intention of promoting increased skeletal changes.^1^ However, the latter has elicited debates and discussions in the orthodontic community, especially in relation to Class II treatments.

These conflicts between one- or two-phase treatments have in some way affected my opinion in relation to interceptive orthodontics and its value. In this edition’s Special Topic, Dr. Marco Antonio Schroeder and co-authors present a segmented mechanics option to treat retained canines, in which they use the posterior segment of the arch as anchorage to preserve more delicate teeth, such as the lateral incisors. The efficiency of interceptive orthodontics in the treatment possibilities of impacted canines also called my attention in this article. The authors state that these interceptive interventions can vary from a simple deciduous canine extraction to a maxillary expansion, and to the use of headgears, all with the purpose of increasing or creating space for spontaneous eruption of the maxillary permanent canines. In fact, just the extraction of deciduous canines in cases of canine retention can increase the chances of eruption of their permanent successors in 50% to 69% of the cases, when compared to controls (36% to 42%).^2^

Not much is known about the true benefits of interceptive orthodontics in the much desired higher level of evidence. And, due to so many possible treatment modalities, when interceptive orthodontics is analyzed as a single procedure and categorized only as the treatment performed before 11 years of age, results of a systematic review demonstrate that this kind of approach does not bring any additional benefits, compared to treatment performed later.^1^ Nevertheless, when these procedures are evaluated separately, results are different. Anterior open bite treatment brings significant dentoalveolar changes in the anterior region, correcting the open bite by incisor extrusion and uprighting. In posterior crossbite cases, results are maintained three years after expansion. Extraction of deciduous canines to reduce anterior crowding does not produce very positive results, with only 15 of 53 patients presenting a crowding reduction greater than 50%. Regarding Class II skeletal changes, there is a significant overjet and ANB reduction, which is not different from those achieved in treatments in the permanent dentition. And, in Class III cases, the use of facemasks increased the ANB, but there is no information of a long term follow up of these results.^1^

Even though it has been demonstrated that teeth, especially projected incisors, are the main cause for bullying among children,^3^ there is very little information on how malocclusions impact quality of life during the mixed dentition. In a recent study, Brazilian children aged 8 to 10 years had the severity of their malocclusion classified by the Dental Aesthetic Index (DAI), while the quality of life was evaluated by the CPQC8-10 questionnaire. Results showed that extremely severe malocclusions, especially a pronounced overjet, have a negative impact on their quality of life.^4^

We are mostly worried with the efficiency of a procedure in the strictly dental aspect, and tend to overlook the additional benefits behind these treatments. The early correction of an increased overjet may not solve a Class II in a definitive manner, but it can have a positive effect on the quality of life of a child, as well as possibly reducing the risk of dental traumas. Also, by only reducing the severity of a malocclusion during an interceptive treatment, we may remove patients from what is considered “medically necessary” to an elective treatment class, as shown by Jolley et al,^5^ who evaluated the effects of interceptive orthodontic treatment in children in the American Medicaid population. 

In this way, with this broadened look and going beyond occlusal benefits, the Brazilian Association of Orthodontics is supporting a law project (PL 2416/2019) which was presented to the Brazilian House of Representatives last April. The project proposes preventive and interceptive orthodontic care in the public health system, with the purpose of promoting self-esteem and psychological well being, essential to the full health of children and teenagers. This law project determines that a specialist in orthodontics should examine once a year children from 6 to 12 years of age in the public health system. In cases that need interceptive treatment, the orthodontist will offer treatment to alleviate these problems. For every 10 elementary schools there will be one orthodontist available. 

It is clear that the interceptive measures should be clearly defined before being performed, since it is well known that, during the mixed dentition, diastemas and irregularities are normal to a certain extent. Otherwise, the benefits here presented will fall into the disadvantages of overtreatment or even malpractice. Well diagnosed interceptive treatments may not reach definitive results, which is our aim under ideal circumstances, but in developing countries such as Brazil, where very little is offered in public health, removing a child from psychological suffering may be a reason for her to smile during her whole life. 

Let’s hope. Good readings!
